# Thrombolysis to Recanalization Time Affects the Benefit of Bridging Thrombolysis in Large Vessel Occlusion Patients With Successful Recanalization

**DOI:** 10.1111/cns.70579

**Published:** 2025-09-14

**Authors:** Gao‐Peng Xing, Wei Li, Chang Cui, Zi‐Ai Zhao, Thanh N. Nguyen, Hui‐Sheng Chen

**Affiliations:** ^1^ Department of Neurology General Hospital of Northern Theater Command Shenyang China; ^2^ Department of Graduate School Dalian Medical University Dalian China; ^3^ Neurology, Radiology Boston Medical Center Boston Massachusetts USA

**Keywords:** endovascular treatment, intravenous thrombolysis, large vessel occlusion, outcome, thrombolysis to recanalization time

## Abstract

**Background and Purpose:**

The benefit of bridging intravenous thrombolysis (IVT) was conflicting in patients with large vessel occlusion (LVO) who received endovascular treatment (EVT). This study aimed to determine whether IVT to recanalization time (TRT) can affect the benefit of IVT bridging EVT.

**Methods:**

Based on a retrospective cohort, eligible LVO patients who achieved successful recanalization after IVT bridging EVT within onset to puncture time of 7 h were enrolled and were divided into TRT ≤ 182 min and TRT > 182 min groups according to median TRT. The primary outcome was the shift in the degree of disability as measured by the modified Rankin Scale (mRS) at 90 days. The primary safety outcome was symptomatic intracranial hemorrhage (sICH). The inverse propensity of treatment weight (IPTW) was used as sensitivity analysis.

**Results:**

A total of 83 eligible patients were enrolled in the final analysis, including 42 in the TRT ≤ 182 min and 41 in the TRT > 182 min group. There was a shift tendency toward a lower degree of functional disability on mRS score at 90 days favoring the TRT ≤ 182 min group compared to the TRT > 182 min group (adjusted OR 1.80, 95% CI 0.69–4.75, *p* = 0.19), which was confirmed by IPTW analysis (OR 1.73, 95% CI 1.16–2.59, *p* = 0.06). A numerically higher proportion of excellent functional outcome at 90 days was found in the TRT ≤ 182 min vs. TRT > 182 min group (56.8% vs. 33.8% before IPTW; 58.4% vs. 25.9% after IPTW). There was no difference in sICH between the TRT ≤ 182 min and TRT > 182 min group.

**Conclusion:**

Among LVO patients who achieved successful recanalization after IVT bridging EVT, the benefit of IVT may be associated with TRT. This finding needs to be validated in prospective trials.

**Trial Registration:**

This trial was registered with ClinicalTrials.gov (NCT04752735)

## Graphical abstract

Based on a retrospective cohort, we determined whether intravenous thrombolysis (IVT) to recanalization time (TRT) can affect the benefit of IVT bridging endovascular treatment (EVT). The results suggest that among LVO patients who achieved successful recanalization after IVT bridging EVT, the benefit of IVT may be associated with TRT.

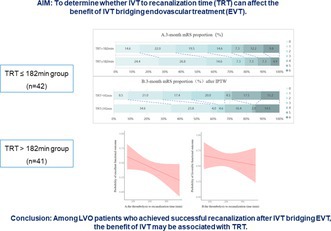

## 1 Introduction

Endovascular treatment (EVT), either alone or following intravenous thrombolysis (IVT), is the standard care for acute ischemic stroke with large vessel occlusion (LVO) [1, 2, 3]. Current guidelines recommend IVT bridging EVT in eligible patients with LVO [4, 5]. However, the benefit of IVT prior to EVT has been controversial considering its relatively poor efficacy in proximal artery recanalization, risk of thrombus fragmentation, and hemorrhagic transformation [6, 7]. Although EVT alone was not shown to be non‐inferior to IVT bridging EVT, the superiority of IVT bridging EVT to EVT alone was also not demonstrated [8, 9, 10, 11, 12, 13, 14]. The benefit associated with IVT bridging EVT versus EVT alone was found to be time dependent and statistically significant only if the time from symptom onset to expected administration of IVT was short [15]. However, the benefit of IVT bridging EVT was observed in real‐world cohorts [16]. In addition, final expanded Thrombolysis in Cerebral Infarction (eTICI) score was significantly improved in patients treated by IVT before EVT [16]. The Endovascular Treatment in Ischemic Stroke (ETIS) registry has shown that in the setting of complete angiographic reperfusion (eTICI 3), patients treated with IVT before EVT had better functional outcomes than those treated with EVT alone [17]. Collectively, these studies suggest the potential benefit of IVT bridging EVT in a time‐dependent manner. However, the underlying mechanisms of the time‐dependent benefit of IVT remain insufficiently understood, which is an important clinical concern. Understanding the mechanism may not only help optimize patient selection for IVT bridging EVT but also refine workflow strategies in acute stroke care.

In light of these data, we hypothesize that the thrombolysis to recanalization time (TRT) may affect the benefit of IVT before EVT through the residual effects of the thrombolytic, which could improve cerebral microcirculation. In this context, this study aimed to test this hypothesis in patients with LVO who achieved successful recanalization after EVT.

## 2 Methods

### 2.1 Patient Selection

DETECT‐China (endovascular treatment for acute ischemic stroke in China: a retrospective, national, multi‐center, registry study) is a retrospective study (NCT04752735), which was approved by the institutional review board of the General Hospital of Northern Theater Command (IRB: y (2021)013) with a waiver of informed consent. From DETECT‐China, we screened consecutive patients with AIS‐LVO who underwent EVT in our stroke center between December 2018 and December 2021. The inclusion criteria were as follows: (1) Age ≥ 18 years; (2) Time from onset to IVT ≤ 4.5 h and time from onset to puncture ≤ 7 h; (3) Successful recanalization (mTICI 2b‐3) after EVT with at least one thrombectomy pass. Patients with missing procedural details, prestroke modified Rankin Scale score (mRS) > 2, missing mRS data, or lack of neuroimaging data were excluded. Based on the median of TRT, eligible patients were divided into TRT ≤ 182 min group and TRT > 182 min group.

### 2.2 Data Collection and Clinical Definitions

The demographic characteristics, stroke risk factors, neurological function scores [NIHSS and mRS], neuroimaging [occlusion site, intracranial hemorrhage (ICH), mTICI, etc.], and procedural details (number of passes, onset to thrombolysis time, onset to puncture time, onset to recanalization time, thrombolysis to puncture time, thrombolysis to recanalization time, etc.) were collected. The primary endovascular strategy included the use of stent retrievers and/or thromboaspiration catheters. Successful recanalization was defined as an mTICI score of 2b–3. Final mTICI score was assessed by two neuroradiologists (Z.A.Z and Y.G.Z) at our center. The clinical outcome was assessed with the mRS at 90 days by certified neurologists through telephone calls or in‐person follow‐up.

### 2.3 Clinical Outcomes

The primary outcome was the shift in the degree of disability as measured by the mRS at 90 days. Secondary outcomes included favorable functional outcome, defined as an mRS 0–2 at 90 days, and excellent functional outcome defined as an mRS 0–1 at 90 days. The primary safety outcome was symptomatic intracranial hemorrhage (sICH), defined as an increase of 4 points or more on the NIHSS within 24 h attributable to intracranial hemorrhage. Other safety outcomes included any intracranial hemorrhage (aICH) and all‐cause mortality at 90 days.

### 2.4 Statistical Analysis

Quantitative variables were expressed as the mean ± standard deviation (SD) for normally distributed parameters or median and interquartile range [IQR] for those with skewed distribution. For normally distributed continuous data, Student's *t*‐tests were used to assess the difference between the two groups. For the non‐normally distributed data, the Mann–Whitney *U*‐test was employed. Categorical variables were presented as number (percentage), and compared using the χ^2^ test, analysis of variance, or Kruskal–Wallis test, as appropriate.

A mRS shift analysis at 90 days was performed with an ordinal logistic model. Then, binary logistic regression analyses were performed for clinical outcome which included excellent and favorable functional outcome at 90 days, the occurrence of 90‐day all‐cause mortality, sICH, and aICH, and presented as odds ratios (OR) with 95% confidence intervals (CI). Comparisons in outcomes were further adjusted for the variables with *p*‐value < 0.25 plus key prognostic covariates.

To account for confounding factors, we used the inverse probability of treatment weighting (IPTW) propensity score method. The propensity score for each individual was defined as the probability of the time from thrombolysis to recanalization in IVT bridging EVT being less than the median, matched for the variables with *p*‐value < 0.25 plus key prognostic covariates. Next, weights were calculated as the inverse of the propensity score and were applied to the study population to create a pseudopopulation. Comparisons in binary outcomes (excellent functional outcome, favorable functional outcome, 90‐day all‐cause mortality, sICH, and aICH) between groups were made using inverse propensity score‐weighted logistic models; odds ratios (ORs) were calculated. Finally, we performed multivariable logistic regression analyses of efficacy and safety outcomes based on the thrombolysis to recanalization time (TRT) (per 10 min) and conducted a correction analysis and restricted cubic splines to predict the relationship between the thrombolysis to recanalization time (TRT) and clinical outcomes. SPSS 26.0 and R software were used for statistical analysis.

## 3 Results

### 3.1 Baseline Characteristics

**FIGURE 1:**
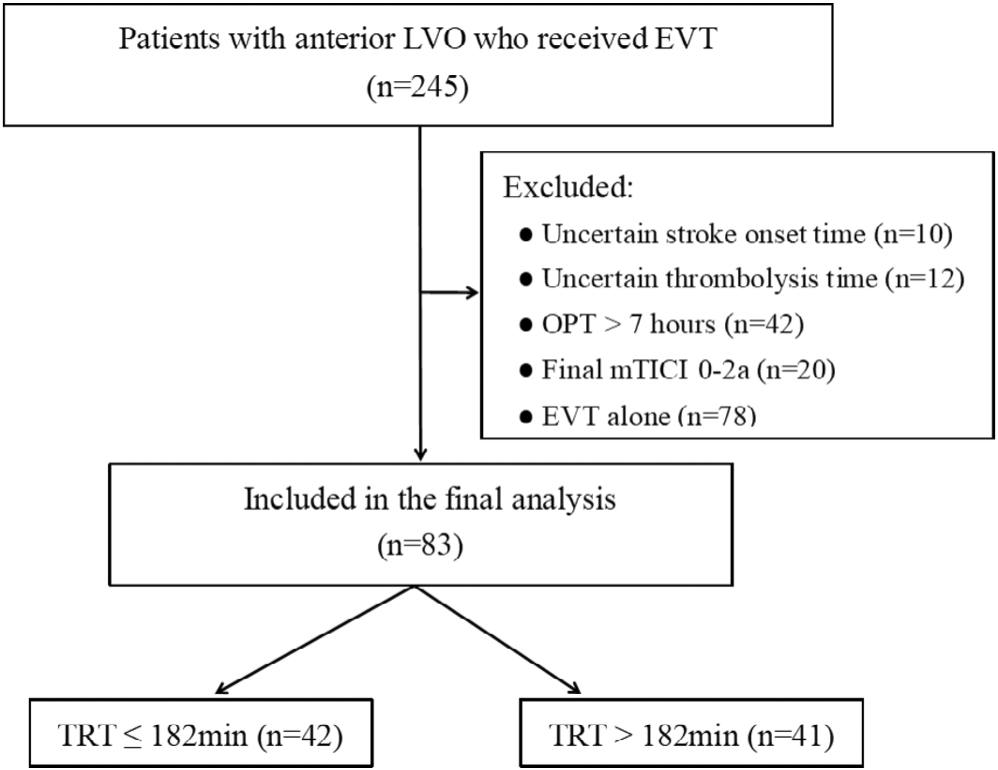
Flowchart of the subject selection. EVT, endovascular treatment; IVT, intravenous thrombolysis; mTICI, modified Thrombolysis in Cerebral Infarction; OPT, onset to puncture time; TRT, thrombolysis to recanalization time.

Among the 245 patients treated with EVT, a total of 83 patients with IVT bridging EVT were enrolled, including 42 (50.6%) with TRT ≤ 182 min and 41 (49.4%) with TRT > 182 min (Figure 1). Table 1 shows baseline characteristics between the TRT ≤ 182 min and TRT > 182 min groups. The baseline characteristics were well balanced between groups except for shorter time from onset to recanalization (ORT) (305 vs. 440 min, *p* < 0.001), onset to puncture time (OPT) (252 vs. 302 min, *p* = 0.005), puncture to recanalization (PRT) (60 vs. 126 min, *p* < 0.001) and thrombolysis to puncture time (TPT) (67 vs. 135 min, *p* < 0.001) in the TRT ≤ 182 min vs. TRT > 182 min group.

**TABLE 1:** Baseline characteristics of patients.

	TRT ≤ 182 min (*n* = 42)	TRT > 182 min (*n* = 41)	*p*
Age, years	66 (55–74)	66 (61–73)	0.895
Sex			
Female	19 (46.3%)	11 (26.8%)	0.081
Male	23 (54.8%)	30 (73.2%)	
Current smoking	17 (40.5%)	22 (53.7%)	0.184
Current drinking	17 (40.5%)	19 (46.3%)	0.503
Past medical history			
Hypertension	27 (64.3%)	28 (68.3%)	0.699
Diabetes	14 (33.3%)	8 (19.5%)	0.154
Ischemic stroke	11 (26.2%)	16 (39.0%)	0.212
Coronary heart disease	9 (21.4%)	9 (22.0%)	0.954
Atrial fibrillation	18 (42.9%)	13 (31.7%)	0.294
Baseline NIHSS	13 (10–16)	13 (9–17)	0.725
NIHSS at 2 weeks or at discharge	5 (2–10)	4 (1–12)	0.760
Prestroke mRS	0	0	1
Presumed stroke cause^a^			
Large artery atherosclerosis	15 (35.7%)	22 (53.7%)	0.259
Cardioembolism	20 (47.6%)	14 (34.1%)	
Other	7 (16.7%)	5 (12.2%)	
Vessel occlusion location			
Middle cerebral artery	29 (69.0%)	26 (63.4%)	0.716
Internal carotid artery	9 (21.4%)	8 (19.5%)	
Basilar artery	3 (7.1%)	4 (9.8%)	
Intravenous thrombolysis			
Alteplase	34 (81.0%)	32 (78.0%)	0.743
Urokinase	8 (19.0%)	9 (22.0%)	
Modified thrombolysis in cerebral infarction			
3	36 (85.7%)	33 (80.5%)	0.364
2b	6 (14.3%)	8 (19.5%)	
Number of passes	1 (1–2)	1 (1–2)	0.207
Endovascular treatment‐related time			
Onset to thrombolysis time	172 (142–217)	163 (143–215)	0.597
Onset to puncture time	252 (191–304)	302 (254–347)	0.005
Onset to recanalization time	305 (266–380)	440 (385–476)	0.000
Puncture to recanalization time	60 (44–84)	126 (84–170)	0.000
Thrombolysis to puncture time	67 (45–94)	135 (82–177)	0.000

*Note:* Data was shown as *N* (%), or median (IQR).

Abbreviations: mRS, modified Rankin Scale; NIHSS, National Institutes of Health Stroke Scale.

^a^ The presumed stroke cause was classified according to the Trial of Org 10,172 in Acute Stroke Treatment (TOAST) classification system.

### 3.2 Clinical Outcomes

**FIGURE 2:**
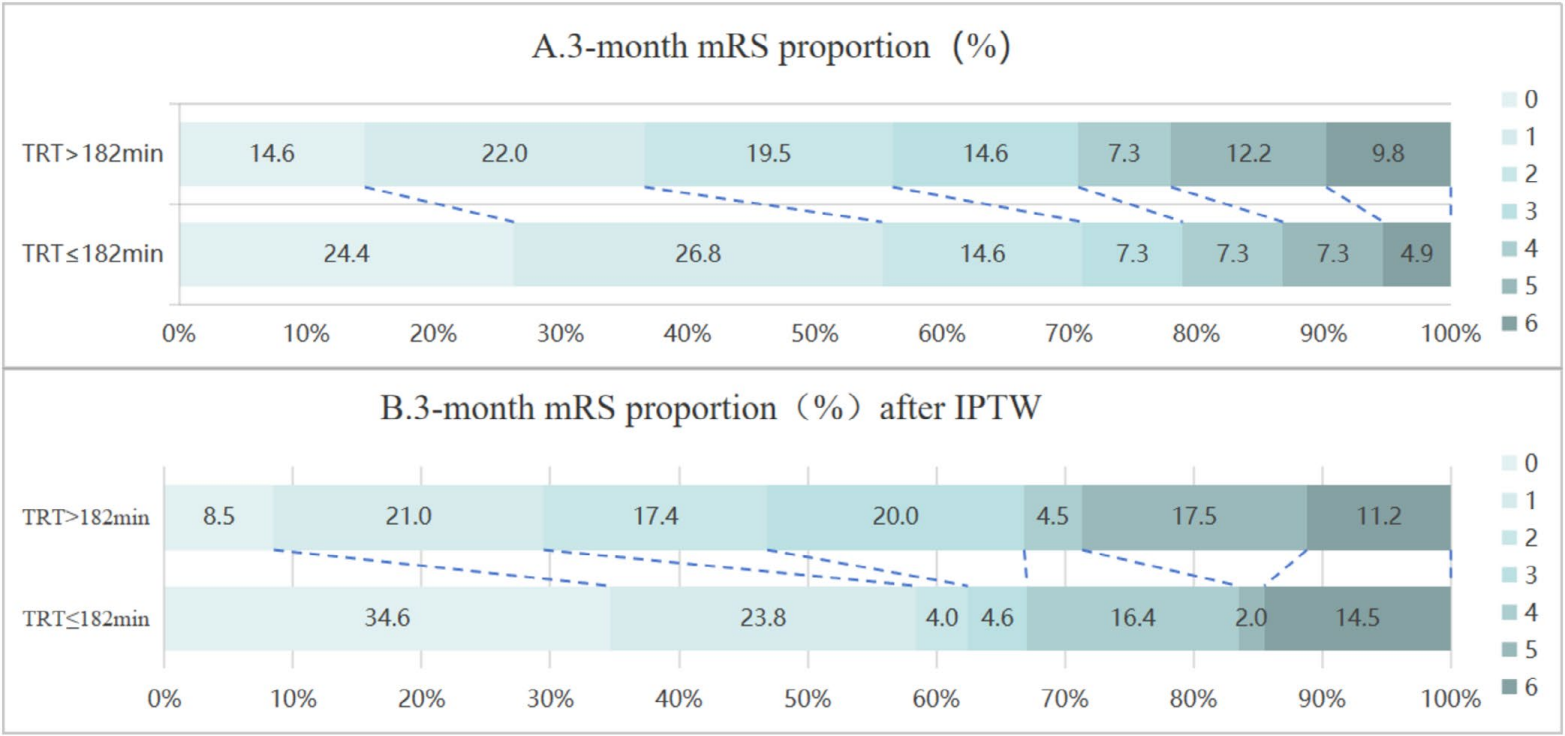
Distribution of modified Rankin Scale (mRS) scores at 90 days.

Figure 2A shows the distribution of mRS scores at 90 days between TRT ≤ 182 min and TRT > 182 min groups. There was a shift tendency toward a lower degree of functional disability on the mRS score at 90 days (1.5 vs. 2, aOR = 1.80, 95% CI = 0.69–4.75, *p* = 0.19) favoring the TRT ≤ 182 min group compared to the TRT > 182 min group. Table 2 presents the clinical outcomes between the TRT ≤ 182 min and TRT > 182 min groups. A numerically higher proportion of excellent (50% vs. 36%) and favorable (64.3% vs. 56.1%) functional outcomes at 90 days was found in the TRT ≤ 182 min group compared with the TRT > 182 min group. There was no significant difference in safety outcomes including mortality (14.6% vs. 9.8%, aOR = 1.31, 95% CI = 0.22–7.80, *p* = 0.77), sICH (4.8% vs. 7.3%, aOR = 1.25, 95% CI = 0.05–29.22, *p* = 0.89), and aICH (24.4% vs. 17.1%, aOR = 4.68, 95% CI = 0.95–22.98, *p* = 0.06). Similar results were found after inverse propensity of treatment weight (IPTW) analysis (Table 3; Figure 2B).

**TABLE 2:** Clinical and safety outcomes.

	TRT ≤ 182 min (*n* = 42)	TRT > 182 min (*n* = 41)	Unadjusted OR (95% CI)	*p*	Adjusted^a^ OR (95% CI)	*p*
mRS distribution at 90 days	1.5 (0.75–4.0)	2 (1–4)	1.39 (0.65–3.00)	0.40	1.80 (0.69–4.75)	0.19
Excellent functional outcome	21 (50.0%)	15 (36.6%)	1.73 (0.72–4.17)	0.22	1.57 (0.47–5.22)	0.46
Favorable functional outcome	27 (64.3%)	23 (56.1%)	1.41 (0.58–3.40)	0.45	2.72 (0.78–9.52)	0.12
Symptomatic intracranial hemorrhage	2 (4.8%)	3 (7.3%)	0.63 (0.10–4.00)	0.63	1.25 (0.05–29.22)	0.89
Any intracranial hemorrhage	10 (24.4%)	7 (17.1%)	1.72 (0.59–5.00)	0.32	4.68 (0.95–22.98)	0.06
Mortality	6 (14.6%)	4 (9.8%)	1.85 (0.50–6.87)	0.36	1.31 (0.22–7.80)	0.77

*Note:* Data was shown as *N* (%), or median (IQR).

Abbreviations: mRS, modified Rankin Scale; OR, odds ratio; TRT, thrombolysis to recanalization time.

^a^ Adjusted for onset to recanalization time, sex, current smoking, diabetes history, history of ischemic stroke, and number of passes.

**TABLE 3:** Clinical and safety outcomes after inverse propensity of treatment weight (IPTW).

	TRT ≤ 182 min (*n* = 453.1)	TRT > 182 min (*n* = 81.6)	OR (95% CI)	*p*
mRS distribution at 90 days	1 (0–4)	2 (1–5)	1.73 (1.16–2.59)	0.06
Excellent functional outcome	264.7 (58.4%)	24.1 (29.5%)	1.84 (0.86–13.05)	0.08
Favorable functional outcome	283.0 (62.5%)	38.3 (46.9%)	3.35 (0.46–7.65)	0.37
Symptomatic intracranial hemorrhage	6.4 (1.4%)	5.0 (6.2%)	0.22 (0.03–1.64)	0.13
Any intracranial hemorrhage	43.3 (9.5%)	13.4 (16.4%)	0.53 (0.13–2.20)	0.38
Mortality	65.5 (14.5%)	9.1 (11.2%)	1.34 (0.23–8.04)	0.74

*Note:* Data was shown as *N* (%), or median (IQR). Inverse propensity of treatment weight matched for onset to recanalization time, sex, current smoking, diabetes history, history of ischemic stroke, and number of passes.

Abbreviations: mRS, modified Rankin Scale; OR, odds ratio; TRT, thrombolysis to recanalization time.

**FIGURE 3:**
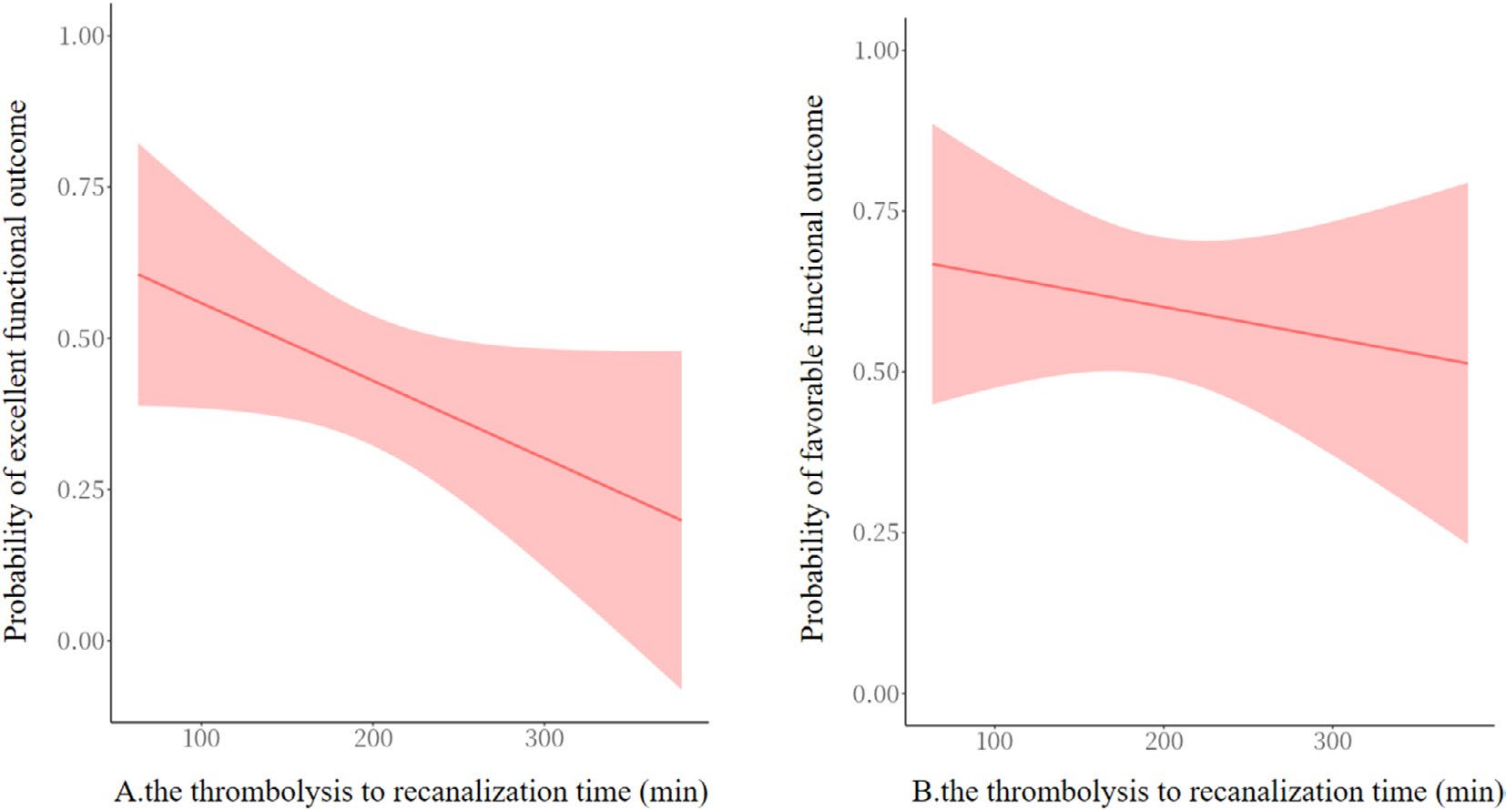
Probability of achieving excellent (A) or favorable functional outcome (B) according to the thrombolysis to recanalization time (TRT).

In addition, we investigated the effect of TRT as a continuous variable on clinical outcomes. After adjusting for ORT, sex, current smoking, diabetes, ischemic stroke, number of passes, the probability of achieving excellent or favorable functional outcome decreased with increasing TRT (Figure 3). TRT was potentially associated with achieving excellent functional outcome (aOR 0.93, 95% CI 0.85–1.01, *p* = 0.10, for every additional 10 min) and favorable functional outcome (aOR 0.93, 95% CI 0.84–1.01, *p* = 0.09, for every additional 10 min). After IPTW, TRT had a significant association with achieving excellent functional outcome (OR 0.87, 95% CI 0.77–0.98, *p* = 0.02, for 10 min) and a potential association with favorable functional outcome (OR 0.92, 95% CI 0.84–1.01, *p* = 0.09).

## 4 Discussion

In this study, we found that functional outcomes potentially improved at 90 days in LVO patients who received IVT bridging EVT and with TRT ≤ 182 min, compared with TRT > 182 min. Furthermore, the probability of achieving good functional outcomes decreased as TRT increased. These results suggest that the benefit of IVT in the setting of EVT may be associated with TRT in patients with LVO stroke; namely, shorter TRT may be associated with a higher probability of good functional outcomes.

A recent meta‐analysis showed that the benefit associated with IVT bridging EVT versus EVT alone was time dependent [15]. In line with these findings, the TIMELESS trial and the CLEAR (CT for Late Endovascular Reperfusion) studies showed no benefit of IVT bridging EVT as compared to EVT alone when patients were treated later [18, 19]. In addition, the phase IIb CHOICE trial suggested that intra‐arterial alteplase may improve clinical outcome in patients with successful recanalization after EVT, which may be attributed to enhanced microcirculatory reperfusion [20, 21]. The recent BRIDGE‐TNK trial showed that tenecteplase (TNK) combined with EVT was superior to EVT alone in patients with LVO stroke within 4.5 h of onset [22], which further supports the concept that intravenous thrombolytics may enhance the efficacy of EVT. In this study, we found that TRT may affect the benefit of intravenous thrombolysis bridging EVT, because shorter TRT may be associated with less probability of disability and increased probability of excellent or favorable functional outcome. Taken together with the IRIS meta‐analysis, the CHOICE results, and the BRIDGE‐TNK results, we propose that the effect of TRT on the benefit of IVT before EVT may be related to the residual thrombolytic effects of thrombolytics on cerebral microcirculation. This proposal was further supported by the fact that effective tissue reperfusion was a stronger predictor of good clinical outcome compared to recanalization [23, 24, 25, 26, 27]. In our study, patients included had a persistent proximal occlusion on the initial angiographic assessment before EVT, aligning with the baseline pre‐IVT imaging, which excluded the direct effect of intravenous thrombolysis on recanalization. The current finding further supports the proposal that microcirculatory dysfunction may be the key mechanism of no‐reflow after EVT, which was consistent with the results from acute myocardial infarction in which microvascular obstruction on cardiac MRI is frequently seen in clinical practice with an incidence of > 50% [28, 29]. A recent study showed that the no‐reflow phenomenon was associated with tissue hypoperfusion despite successful recanalization [27, 30].

The key strength of this study is that it provides the first possible insight that TRT may affect the benefit of IVT before EVT through the residual thrombolytic effects of alteplase on the cerebral microcirculation. This finding may partially explain the controversy between EVT alone and IVT bridging EVT and provide a possible reference for future clinical trials. However, we acknowledge several limitations. The main limitation is a retrospective single‐center analysis with a relatively small sample size, thus inevitably leading to confounding bias, affecting the statistical power, and limiting the generalizability of our findings. To minimize this limitation, we performed IPTW as a sensitivity analysis; similar results were found after IPTW. Second, most enrolled patients were M1 occlusions (68.8%), which limits the application of this finding to other sites of LVO (internal carotid artery or M2). Third, systematic perfusion imaging was not performed after successful angiographic reperfusion by EVT. Therefore, this study cannot fully demonstrate that the positive effect of IVT is driven by improved microvascular reperfusion. As intravenous tenecteplase is being used more commonly, it is unknown how our findings will translate with newer or other thrombolytics. Finally, this finding should be validated in non‐Chinese patients given the differences in co‐morbid factors and etiology of LVO when compared with other populations. Further multicenter, prospective studies with larger sample sizes, multi‐ethnic cohorts, and standardized imaging protocols are needed to validate and extend our findings.

## 5 Conclusion

Among LVO patients who achieved successful recanalization after IVT bridging EVT, the benefit of IVT may be associated with TRT. This finding needs to be validated in prospective trials.

## Author Contributions

G.‐P.X. W.L. and C.C. contributed to the acquisition and analysis of data. G.‐P.X. and W.L. drafted the first manuscript. T.N.N. critically revised the manuscript. H.‐S.C. designed the study and critically revised the manuscript.

## Conflicts of Interest

Thanh N. Nguyen reports as Associate Editor of Stroke; Advisory board of Brainomix, Aruna Bio; Speaker for Genentech, Kaneka; consulting for Medtronic.

## Data Availability

Data are available upon reasonable request to the corresponding author.

## References

[1] M. Goyal , B. K. Menon , W. H. van Zwam , et al., “Endovascular Thrombectomy After Large‐Vessel Ischaemic Stroke: A Meta‐Analysis of Individual Patient Data From Five Randomised Trials,” Lancet 387, no. 10029 (2016): 1723–1731, 10.1016/S0140-6736(16)00163. 26898852

[2] T. N. Nguyen , M. Abdalkader , U. Fischer , et al., “Endovascular Management of Acute Stroke,” Lancet 404, no. 10459 (2024): 1265–1278, 10.1016/S0140-6736(24)01410-7. 39341645

[3] C. J. Winstein , J. Stein , R. Arena , et al., “Guidelines for Adult Stroke Rehabilitation and Recovery: A Guideline for Healthcare Professionals From the American Heart Association/American Stroke Association,” Stroke 47, no. 6 (2016): e98–e169, 10.1161/STR.0000000000000098. 27145936

[4] G. Turc , G. Tsivgoulis , H. J. Audebert , et al., “European Stroke Organisation (ESO)‐European Society for Minimally Invasive Neurological Therapy (ESMINT) Expedited Recommendation on Indication for Intravenous Thrombolysis Before Mechanical Thrombectomy in Patients With Acute Ischemic Stroke and Anterior Circulation Large Vessel Occlusion,” Journal of Neurointerventional Surgery 14, no. 3 (2022): 209, 10.1136/neurintsurg-2021-018589I. 35115395

[5] H. E. Masoud , A. de Havenon , A. C. Castonguay , et al., “Brief Practice Update on Intravenous Thrombolysis Before Thrombectomy in Patients With Large Vessel Occlusion Acute Ischemic Stroke: A Statement From Society of Vascular and Interventional Neurology Guidelines and Practice Standards (GAPS) Committee,” Stroke: Vascular and Interventional Neurology 2 (2022): e000276. 10.1161/SVIN.121.000276 PMC12778797 41584777

[6] P. Seners , G. Turc , B. Maïer , J. L. Mas , C. Oppenheim , and J. C. Baron , “Incidence and Predictors of Early Recanalization After Intravenous Thrombolysis: A Systematic Review and Meta‐Analysis,” Stroke 47, no. 9 (2016): 2409–2412, 10.1161/STROKEAHA.116.014181. 27462117

[7] J. Emberson , K. R. Lees , P. Lyden , et al., “Effect of Treatment Delay, Age, and Stroke Severity on the Effects of Intravenous Thrombolysis With Alteplase for Acute Ischaemic Stroke: A Meta‐Analysis of Individual Patient Data From Randomised Trials,” Lancet 384, no. 9958 (2014): 1929–1935, 10.1016/S0140-6736(14)60584-5. 25106063 PMC4441266

[8] P. Yang , Y. Zhang , L. Zhang , et al., “Endovascular Thrombectomy With or Without Intravenous Alteplase in Acute Stroke,” New England Journal of Medicine 382, no. 21 (2020): 1981–1993, 10.1056/NEJMoa2001123. 32374959

[9] U. Fischer , J. Kaesmacher , D. Strbian , et al., “Thrombectomy Alone Versus Intravenous Alteplase Plus Thrombectomy in Patients With Stroke: An Open‐Label, Blinded‐Outcome, Randomised Non‐Inferiority Trial,” Lancet 400, no. 10346 (2022): 104–115, 10.1016/S0140-6736(22)00537-2. 35810756

[10] P. J. Mitchell , B. Yan , L. Churilov , et al., “Endovascular Thrombectomy Versus Standard Bridging Thrombolytic With Endovascular Thrombectomy Within 4·5 h of Stroke Onset: An Open‐Label, Blinded‐Endpoint, Randomised Non‐Inferiority Trial,” Lancet 400, no. 10346 (2022): 116–125, 10.1016/S0140-6736(22)00564-5. 35810757

[11] K. Suzuki , Y. Matsumaru , M. Takeuchi , et al., “Effect of Mechanical Thrombectomy Without vs With Intravenous Thrombolysis on Functional Outcome Among Patients With Acute Ischemic Stroke: The SKIP Randomized Clinical Trial,” Journal of the American Medical Association 325, no. 3 (2021): 244–253, 10.1001/jama.2020.23522. 33464334 PMC7816103

[12] N. E. LeCouffe , M. Kappelhof , K. M. Treurniet , et al., “A Randomized Trial of Intravenous Alteplase Before Endovascular Treatment for Stroke,” New England Journal of Medicine 385, no. 20 (2021): 1833–1844, 10.1056/NEJMoa2107727. 34758251

[13] W. Zi , Z. Qiu , F. Li , et al., “Effect of Endovascular Treatment Alone vs Intravenous Alteplase Plus Endovascular Treatment on Functional Independence in Patients With Acute Ischemic Stroke: The DEVT Randomized Clinical Trial,” Journal of the American Medical Association 325, no. 3 (2021): 234–243, 10.1001/jama.2020.23523. 33464335 PMC7816099

[14] C. B. Majoie , F. Cavalcante , J. Gralla , et al., “Value of Intravenous Thrombolysis in Endovascular Treatment for Large‐Vessel Anterior Circulation Stroke: Individual Participant Data Meta‐Analysis of Six Randomised Trials,” Lancet 402, no. 10406 (2023): 965–974, 10.1016/S0140-6736(23)01142-X. 37640037

[15] J. Kaesmacher , F. Cavalcante , M. Kappelhof , et al., “Time to Treatment With Intravenous Thrombolysis Before Thrombectomy and Functional Outcomes in Acute Ischemic Stroke: A Meta‐Analysis,” Journal of the American Medical Association 331, no. 9 (2024): 764–777, 10.1001/jama.2024.0589. 38324409 PMC10851137

[16] Y. Wang , X. Wu , C. Zhu , M. Mossa‐Basha , and A. Malhotra , “Bridging Thrombolysis Achieved Better Outcomes Than Direct Thrombectomy After Large Vessel Occlusion: An Updated Meta‐Analysis,” Stroke 52, no. 1 (2021): 356–365, 10.1161/STROKEAHA.120.031477. 33302795

[17] B. Gory , S. Finitsis , J. M. Olivot , et al., “Intravenous Thrombolysis Before Complete Angiographic Reperfusion: Beyond Angiographic Assessment to Target Microvascular Obstruction?,” Annals of Neurology 95, no. 4 (2024): 762–773, 10.1002/ana.26867. 38148607

[18] G. W. Albers , M. Jumaa , B. Purdon , et al., “Tenecteplase for Stroke at 4.5 to 24 Hours With Perfusion‐Imaging Selection,” New England Journal of Medicine 390, no. 8 (2024): 701–711, 10.1056/NEJMoa2310392. 38329148

[19] J. Demeestere , M. M. Qureshi , L. Vandewalle , et al., “Outcomes of Bridging Intravenous Thrombolysis Versus Endovascular Therapy Alone in Late‐Window Acute Ischemic Stroke,” Stroke 55, no. 7 (2024): 1767–1775, 10.1161/STROKEAHA.124.046495. 38748598

[20] A. Renú , M. Millán , L. San Román , et al., “Effect of Intra‐Arterial Alteplase vs Placebo Following Successful Thrombectomy on Functional Outcomes in Patients With Large Vessel Occlusion Acute Ischemic Stroke: The CHOICE Randomized Clinical Trial,” Journal of the American Medical Association 327, no. 9 (2022): 826–835, 10.1001/jama.2022.1645. 35143603 PMC8832304

[21] P. Khatri , “Intra‐Arterial Thrombolysis to Target Occlusions in Distal Arteries and the Microcirculation,” Journal of the American Medical Association 327, no. 9 (2022): 821–823, 10.1001/jama.2021.25014. 35143600

[22] Z. Qiu , F. Li , H. Sang , et al., “Intravenous Tenecteplase Before Thrombectomy in Stroke,” New England Journal of Medicine 393, no. 2 (2025): 139–150, 10.1056/NEJMoa2503867. 40396577

[23] B. P. Soares , E. Tong , J. Hom , et al., “Reperfusion Is a More Accurate Predictor of Follow‐Up Infarct Volume Than Recanalization: A Proof of Concept Using CT in Acute Ischemic Stroke Patients,” Stroke 41, no. 1 (2010): e34–e40, 10.1161/STROKEAHA.109.568766. 19910542 PMC2909663

[24] T. H. Cho , N. Nighoghossian , I. K. Mikkelsen , et al., “Reperfusion Within 6 Hours Outperforms Recanalization in Predicting Penumbra Salvage, Lesion Growth, Final Infarct, and Clinical Outcome,” Stroke 46, no. 6 (2015): 1582–1589, 10.1161/STROKEAHA.114.007964. 25908463

[25] M. Rubiera , A. Garcia‐Tornel , M. Olivé‐Gadea , et al., “Computed Tomography Perfusion After Thrombectomy: An Immediate Surrogate Marker of Outcome After Recanalization in Acute Stroke,” Stroke 51, no. 6 (2020): 1736–1742, 10.1161/STROKEAHA.120.029212. 32404034

[26] D. A. De Silva , J. N. Fink , S. Christensen , et al., “Assessing Reperfusion and Recanalization as Markers of Clinical Outcomes After Intravenous Thrombolysis in the Echoplanar Imaging Thrombolytic Evaluation Trial (EPITHET),” Stroke 40, no. 8 (2009): 2872–2874, 10.1161/STROKEAHA.108.543595. 19478228

[27] F. C. Ng , L. Churilov , N. Yassi , et al., “Prevalence and Significance of Impaired Microvascular Tissue Reperfusion Despite Macrovascular Angiographic Reperfusion (No‐Reflow),” Neurology 98, no. 8 (2022): e790–e801, 10.1212/WNL.0000000000013210. 34906976

[28] T. Dalkara , “Pericytes: A Novel Target to Improve Success of Recanalization Therapies,” Stroke 50, no. 10 (2019): 2985–2991, 10.1161/STROKEAHA.118.023590. 31495330

[29] R. A. Kloner , K. S. King , and M. G. Harrington , “No‐Reflow Phenomenon in the Heart and Brain,” American Journal of Physiology. Heart and Circulatory Physiology 315, no. 3 (2018): H550–H562, 10.1152/ajpheart.00183.2018. 29882685

[30] C. A. Mutimer , A. Mujanovic , J. Kaesmacher , et al., “Comparison of Perfusion Imaging Definitions of the No‐Reflow Phenomenon After Thrombectomy‐What Is the Best Perfusion Imaging Definition?,” Annals of Neurology 96 (2024): 1104–1114, 10.1002/ana.27073. 39225109

